# Supplementary material for: Adenine Base Editing Potently Suppresses Hepatitis B Surface Antigen Expression and Inhibits Hepatitis D Virus Release
Source: bioRxiv. 2026 Feb 6:2026.02.06.704371. Preprint. [Version 1] doi: 10.64898/2026.02.06.704371 (PMC12889661; doi:10.64898/2026.02.06.704371)
Supplement: Supplement 1 [file media-1.pdf]

Supplementary Fig. S1

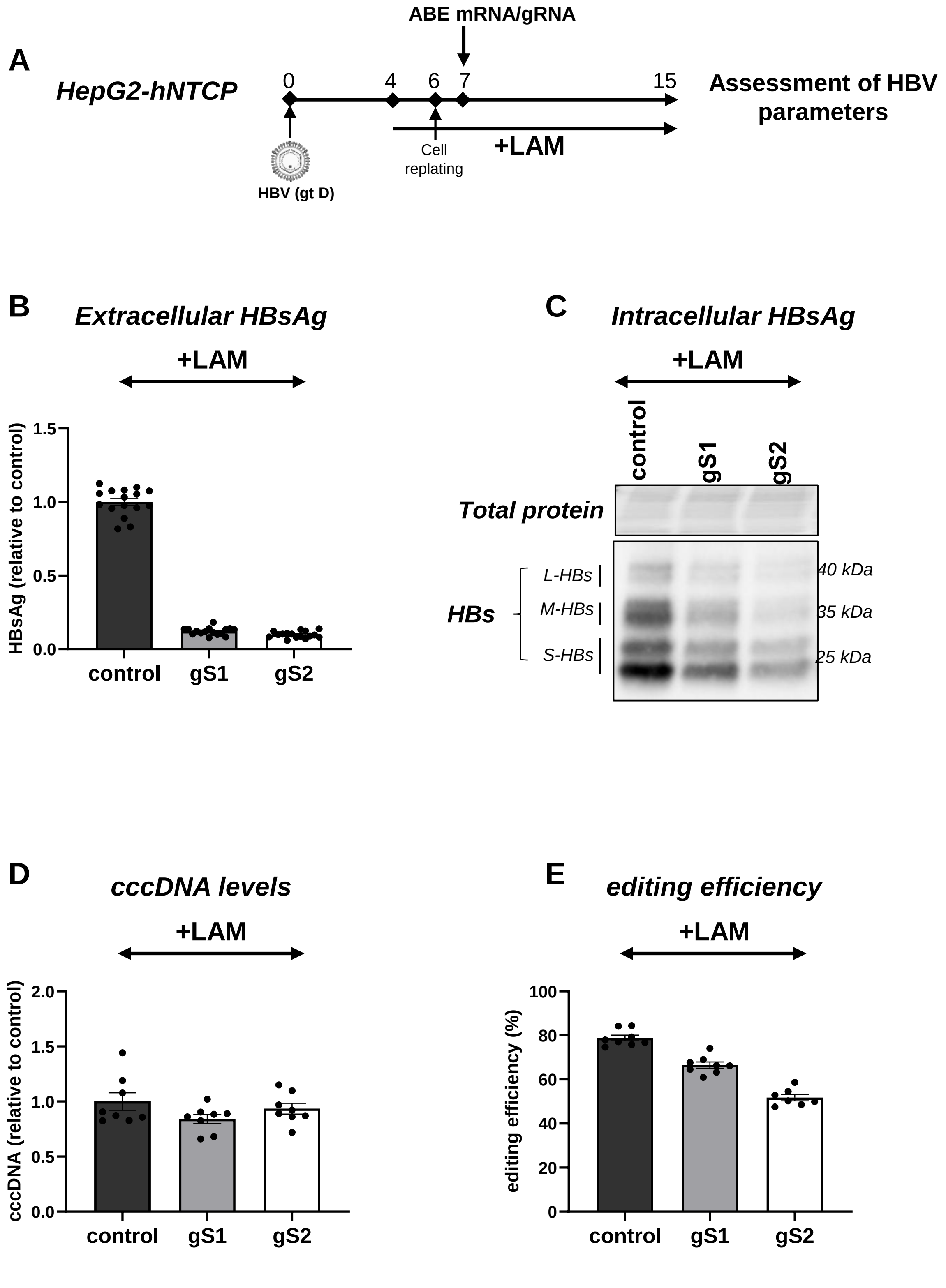

Fig S1 Adenine base editing can be combined with NA. (A) The schematic of the protocol used to transfect Lamivudine (LAM) pretreated HBV-infected HepG2-hNTCP cells with ABE-encoding mRNA and gS1 or gS2. (B-C) The levels of extracellular HBsAg and intracellular HBsAg were determined by ELISA and Western blot, respectively. (D) cccDNA levels were quantified by qPCR. (E) Percentage of A-to-G editing of HBs/POL ORF by gS1 and gS2 and control was determined on exonucleases I/III-treated samples and total extracted DNA samples, respectively. control: non-HBV targeting gRNA; LAM: Lamivudine. Data are represented as mean±SEM.

Supplementary Fig. S2

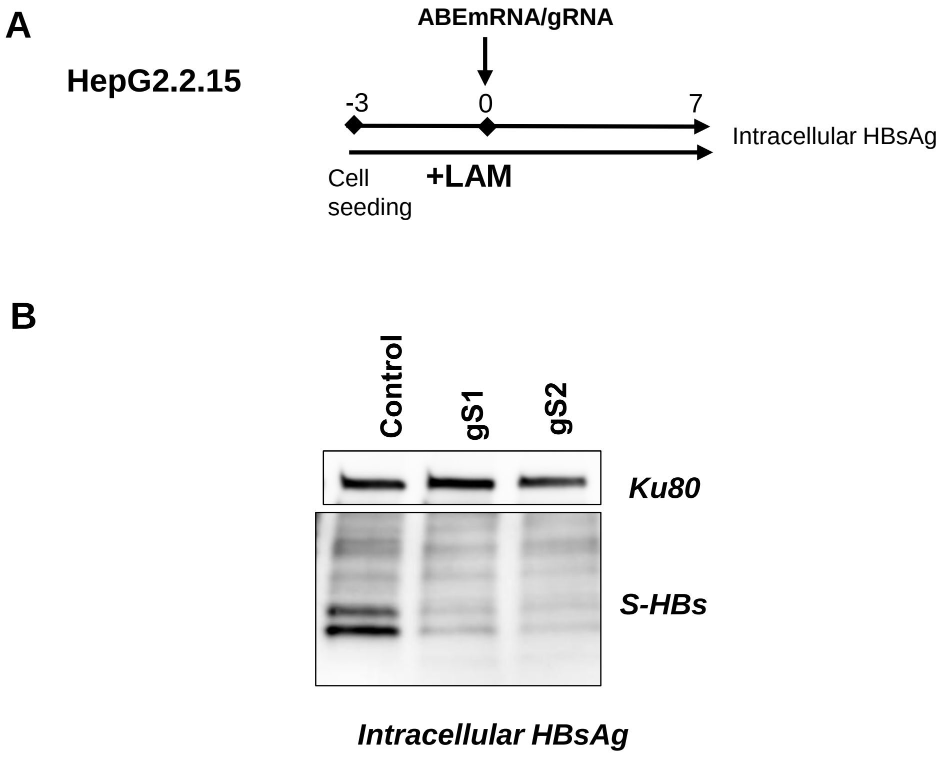

Fig S2: ABE/gRNAs drastically reduce intracellular HBsAg from integrated HBV DNA (A) The schematic of the protocol used to transfect LAM pretreated HepG2.2.15 cells with ABE-encoding mRNA and gS1 or gS2. (B) The levels of intracellular HBsAg was determined by Western blot. control gRNA: non-HBV targeting gRNA; LAM: Lamivudine

Supplementary Fig. S3

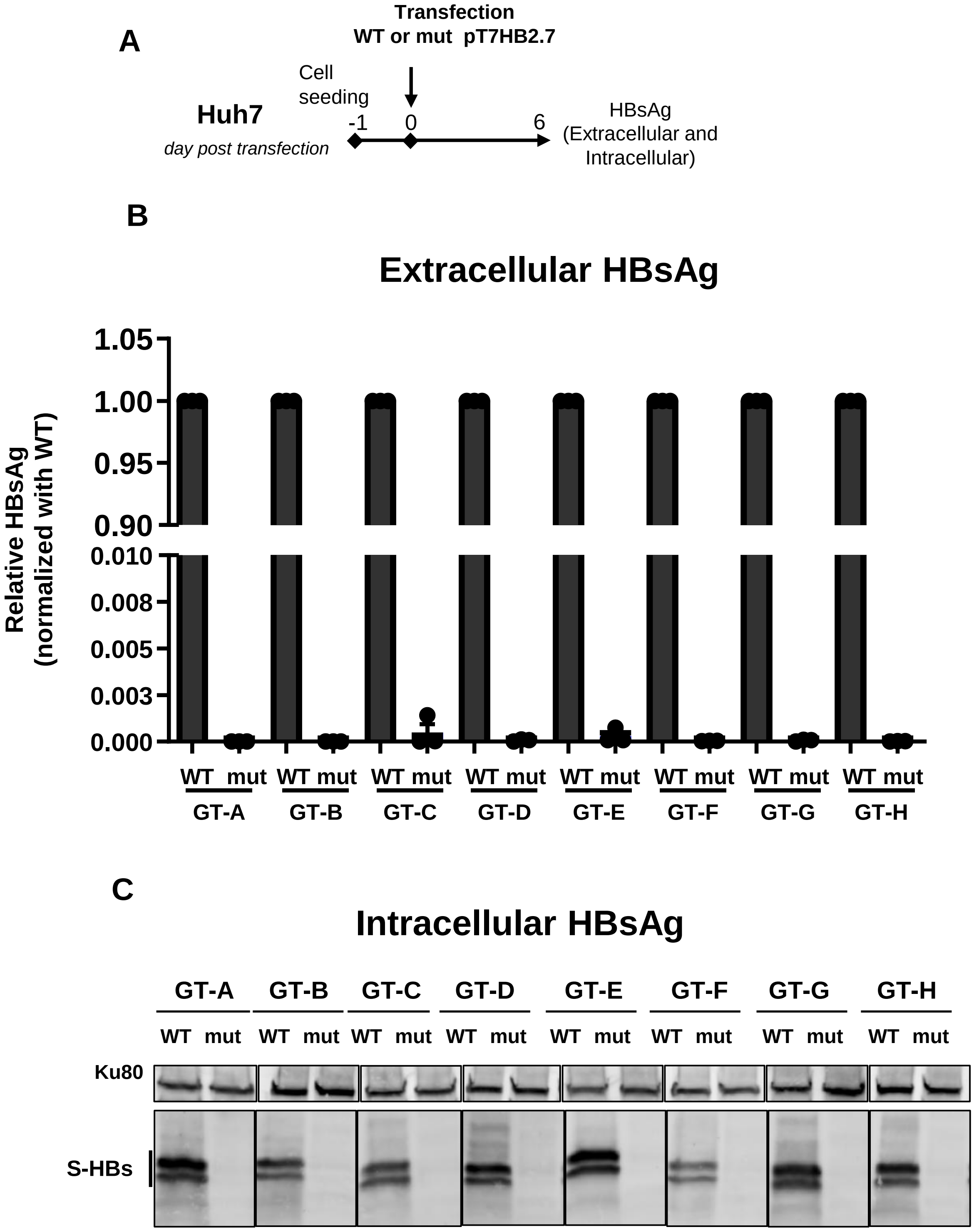

Fig. S3. (A) Experimental scheme (B-C) ELISA and Western blots showing the effect of gS2-corresponding mutations on secreted and intracellular HBsAg levels, respectively, across different HBV genotypes. Data were normalized with respect to WT condition for each genotype. (WT: wildtype; mut: mutant containing gS2-corresponding mutations; GT: HBV genotype; Letters A to H represent HBV genotypes)

Supplementary Fig. S4

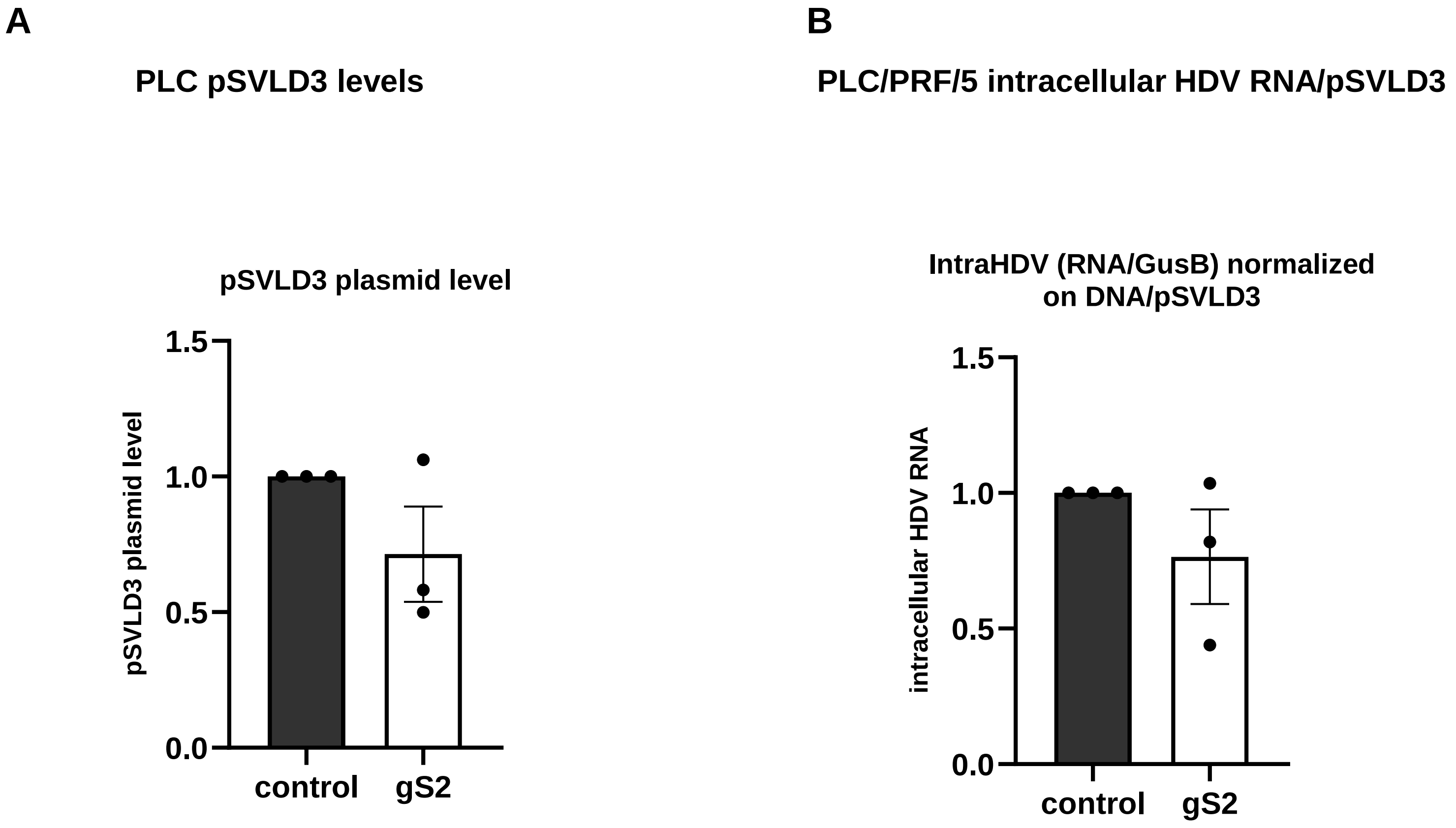

Fig. S4. For the experiment in Fig. 6, intracellular (A) pSVLD3 plasmid (B) HDV RNA levels were quantified from PLC/PRF/5 cells. The data were normalized with respect to control gRNA condition.

Supplementary Fig. S5

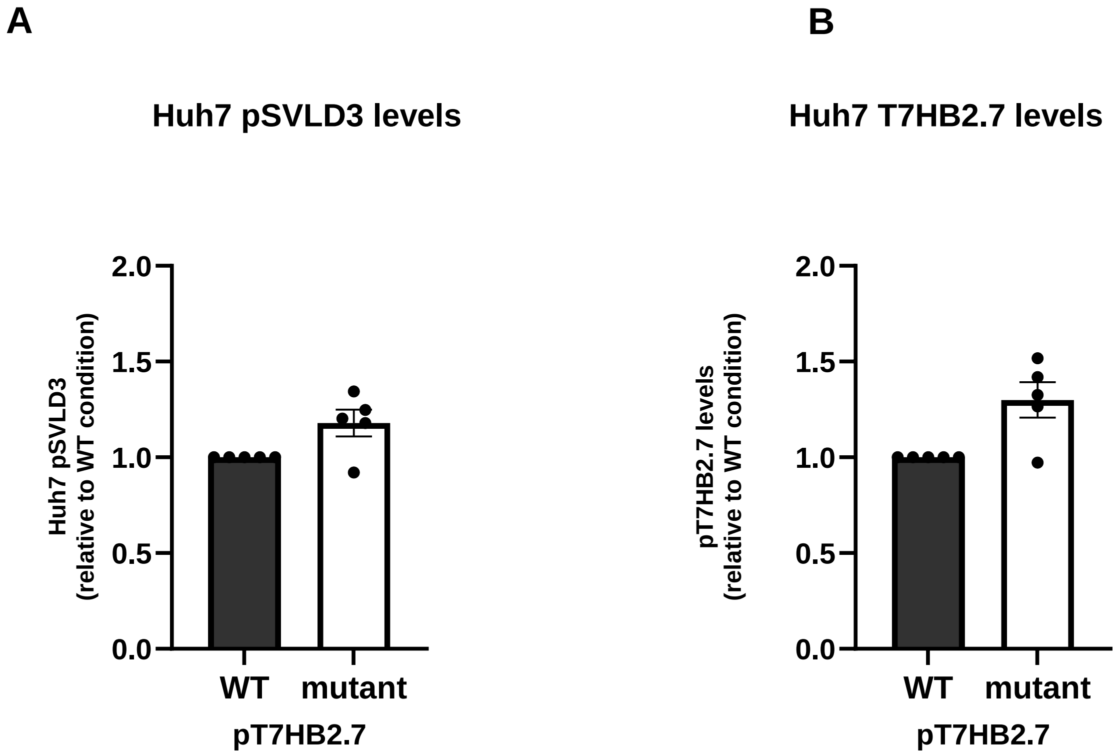

Fig. S5. For the experiment in Fig. 7, intracellular (A) pSVLD3 and (B) pT7HB2.7 plasmid levels were quantified from Huh7 cells. The data were normalized with respect to pT7HB2.7-WT condition.
